# Ethical issues in microbiome research and medicine

**DOI:** 10.1186/s12916-016-0702-7

**Published:** 2016-10-12

**Authors:** Rosamond Rhodes

**Affiliations:** Icahn School of Medicine at Mount Sinai, New York, NY USA

**Keywords:** Microbiome, Ethics, Research ethics, Public health, Biobanks, Privacy, Property

## Abstract

The human microbiome is the collection of bacteria, viruses, and fungi that live on and in the human organism’s skin, mucosa, and intestinal tract. Re-examining commonly accepted ethical standards from the perspective of this new area of research provides an opportunity to reassess our current thinking about research regulations as well as the importance of some principles and distinctions. In this commentary, I explain ethical issues illuminated by research on the human microbiome related to personal identity, privacy, property, research ethics, public health, and biobanks.

## Background

The cohabitation of the human genome and the genomes of the bacteria and viruses that occupy our skin, mucous membranes, intestinal tract, and other parts of our bodies together make up the microbiome. In 2007, the National Institute of Health launched the Human Microbiome Project to utilize technological advances to characterize the microbial communities that inhabit the human body and explore the relationships between the microbiota and their human hosts, including the effect that they may have on human health and disease, development, physiology, immunity, and nutrition.

Learning about the microbiome will change how medicine is practiced. It may also have implications for our social and legal systems and for how we conceive the ethics of medicine and biomedical research. Therefore, it is important to identify the ethical, legal, and social implications raised by human microbiome research in order to advise both the scientists engaged in the work and members of society who will participate in studies and live with the consequences .

Addressing issues from the vantage point of microbiome research provides a fresh perspective on who we are, our place in the world, and our responsibilities to one another. For example, research on the human microbiome calls for a paradigm shift from thinking about germs as enemies that must be hunted and destroyed to thinking about achieving a healthy microbiotic environment around and within us. Further, clinicians and investigators will be expanding their focus from diagnosing individual genetic anomalies to developing an understanding of the human genome and its interactions with the microbiome [1–3]. In addition, studies to advance personalized medicine will require broad public participation to provide sufficient material for biobanks and sample banks rather than a small sample of people with a target condition. Although personhood and identity have never been simple concepts, as we learn more about ourselves as an amalgam of us and the microbes that live on us and within us, we will rethink our concepts of personal identity and normalcy. Thus, in numerous ways, learning about the microbiome may shift the moral perspective from a focus on individual rights and liberties toward a community perspective that values solidarity.

## Personal identity

“I” used to be a simple term, and everyone knew what “I” meant. Now, in light of what we are learning from science, “I” refers to me as a moral agent, and the subject of my consciousness, and my genotype, and my phenotype, and my microbiome comprised of critters that are not me, many of which come and go. In different contexts, different concepts of “me” are relevant. Ethically, only some humans are moral persons, because only some can be held responsible for their actions (e.g., young children cannot be). As a victim of disease, my body is my identity. As a vector of disease, my microbiome is my identity. The microbiome has an impact on the health of the human organism, but its effects are determined, in part, by the combined characteristics of the microbes that comprise it. A particular species of microbe might have positive effects in one human and negative effects in another. These differences will complicate efforts to define what is “normal.” Although personhood and identity have never been simple concepts, as we come to see ourselves as an amalgam of us and them, we will have to rethink concepts of personal identity, normalcy, and what they imply [4, 5].

Our understanding of the human microbiome and its interaction with the human body also has implications for how we conceptualize both personhood and personal identity. Personhood is usually defined in terms of essential and distinguishing characteristics. Thus, if genes, diet, and microbes distinguish our susceptibility and resistance to disease and responsiveness to treatments, they may all be part of our identity [6, 7]. Furthermore, each individual’s microbiome is unique [8]. In that sense, the microbiome may be incorporated into how we define ourselves as persons.

## Privacy

For the most part, the borders of privacy conform to physical boundaries. They coincide with personal enclosed spaces such as my body, my home, behind the closed door of my bedroom, inside my own diary, and inside my own thoughts. With few exceptions, no one may enter my private domain without my permission, and governmental intrusions require robust justification.

Confidentiality is different from privacy [9]. Confidentiality is an important professional responsibility for clinicians and some other professionals (e.g., lawyers, accountants, priests). In these professions, an artificial space is created within which information is safeguarded. Within those boundaries, information disclosed by those seeking professional services may be shared in order to promote the client’s interests. Outside of those boundaries, disclosed information may be divulged only with the client’s permission. Thus, in medicine, patients expect their medical history, diagnosis, and prognosis only to be shared among the health professionals who need it for providing care. Beyond that, patients reasonably expect their information not to be divulged. The assurance of confidentiality is critical for the practice of medicine because it allows patients to freely share information.

Although the concepts of “confidentiality” and “privacy” are frequently conflated and the distinctions elided, the difference may be critical in work on the human microbiome. When we examine privacy and confidentiality from the perspective of research on the microbiome we will have to consider which information should be protected and why. Many research ethics discussions come down strongly on the side of treating research samples with privacy protections, but at this point it is appropriate to ask whether strict limitations on sharing samples are reasonable.

Learning about the human microbiome will raise additional privacy questions with important implications in matters of personal liberty. Approximately 95 % of my feces are microbiome, and my microbiome tells the story of where I have been and with whom I have associated. Should law enforcement agents have access to my microbiome in the same way that they are allowed to collect my fingerprint trail?

## Property

In the philosophical literature, we see radically different views on property, and the ethical, legal, and social questions about who owns what. These controversies extend to deposits in biobanks and sample banks [10, 11]. Do we have property rights in our microbiome because our body is its host? Under some circumstances the government may mandate invasions into (e.g., vaccinations) and uses of (e.g., compelled military service) our bodies. May government rightfully demand that we contribute genome or microbiome samples to research or public health activities? When knowledge from a microbiome study has commercial value, who should reap the profits? If someone’s microbiome is unique and valuable, who owns it? Although we have not yet seen lawsuits involving the human microbiome, there have recently been a few legal rulings related to biological samples and genetic research that are relevant to the human microbiome.

## Human subject research

According to popular accounts of research ethics history [12, 13], the development of research standards and regulations begins with the Nuremberg Code, followed by the World Medical Association’s Helsinki Agreement [14, 15]. The second half of the twentieth century was also the period of the assertion of civil rights, women’s rights, and rights of people with disabilities, all of which are movements argued in terms of autonomy. It is no surprise, therefore, that The Belmont Report and the Common Rule, the US research regulations that it produced, as well as the International Ethical Guidelines for Biomedical Research Involving Human Subjects are informed by that concept and expressed in terms of respect for persons [16, 17]. That commitment to autonomy gives primacy to informed consent and privacy in contemporary research ethics.

In this tradition of research ethics, the authority to employ human subjects in studies derives from the participant’s informed consent. The decision to participate in a study is taken as endorsement of the research goals and as free acceptance of the burdens and risks involved as being reasonable under the circumstances. In keeping with the twentieth century’s focus on autonomy, a good deal of the NIH Human Genome Project work on the ethical, legal, and social implications of research on the human genome has focused on individual liberty- and autonomy-related human rights concerns.

At the same time, the literature on the ethical, legal, and social implications of the human genome study has largely overlooked what John Gray has described as *the other face of social contract theory, the obligations of the participant* [18]. Yet, at this stage in the development of the science, researchers have expanded their focus from the diagnosis of individual genetic anomalies to the broader issues of understanding the human genome and its interactions with the microbiome, for example, in diabetes and inflammatory bowel diseases. As many have noted, studies to advance personalized medicine will require broad participation to provide the material for biobanks and sample banks [19, 20]. These efforts will raise issues of whether we should all contribute to the enterprise. In that some genetic and microbiome research is supported by public funds, we all contribute our financing to the efforts. Should we also contribute bits of ourselves, our genomes, and our microbiomes? If we expect to benefit from what is learned, but are unwilling to participate, will we be free-riders on the altruism of others? Will we be treating others unjustly? Answering these questions will require clarification of the individual responsibilities of members of society.

## Public health

Similar issues arise in public health and public health research [21, 22]. Because the microbiome is a factor in some infectious diseases, protecting public health requires disease surveillance and tracking. Data collection on disease outbreaks, births, and deaths is and will be critical in the design and implementation of effective disease prevention, outbreak control, and disaster response. Effective public health measures are based on data, but gathering personal information in an emergency may not allow for prior Institutional Review Board assessment or informed consent [23].

Most clinical research exposes only individual subjects to risks and harms. Yet, as research on the human microbiome leads to studies of prebiotic, probiotic (bacterial), and phage (viral) therapies, the risks to the public as well as research subjects will be relevant considerations. When we contemplate altering the human microbiome by inducing changes in naturally occurring collections of microbes, we need to be cognizant of the fact that people are both vectors and victims of disease [24, 25]. Furthermore, microbial agents that are beneficial to some humans may be toxic to other humans and other organisms. Additionally, because microbes that may benefit some may be communicated to others and harm them, we will have to evaluate and assess the complex potential public health risks associated with studies of conditions such as obesity and asthma [26].

### Biobanks

It is easy to appreciate that we all want to avoid diseases and their burdens and, when these cannot be avoided, we expect efficient and effective means to overcome them. Having such treatments available will require significant scientific advances that necessitate basic science and translational research involving biobanking, sample banking, and human subject research. For example, the goal of advancing the treatment of cancer through personalized medicine requires research on samples from a large number of individuals with different cancer types [27–29]. The emerging need for further research and more general cooperation in research raises questions about the rules and governance of biobank and sample banks. Who should be given access to the biological samples, which information should be shared, which projects should the samples support, and who should make these decisions?

## Conclusion

The use of technology employing knowledge of the human microbiome is just getting started and is not yet widely applied. Nevertheless, it is important to consider the ethical issues early on so that we may try to avoid at least those pitfalls that can be identified now rather than waiting for disasters to occur and then trying to cobble together hasty responses. Fresh consideration of these issues by the biomedical research community, eschewing both exaggeration of the risks and ignoring real issues, will require both clear thinking and courage.
